# Monkeypox detection using deep neural networks

**DOI:** 10.1186/s12879-023-08408-4

**Published:** 2023-06-27

**Authors:** Amir Sorayaie Azar, Amin Naemi, Samin Babaei Rikan, Jamshid Bagherzadeh Mohasefi, Habibollah Pirnejad, Uffe Kock Wiil

**Affiliations:** 1 Department of Computer Engineering, Urmia University, Urmia, Iran; 2 Center for Health Informatics and Technology, The Maersk Mc-Kinney Moller Institute, University of Southern Denmark, Odense, Denmark; 3 Patient Safety Research Center, Clinical Research Institute, Urmia University of Medical Sciences, Urmia, Iran; 4 Erasmus School of Health Policy & Management (ESHPM), Erasmus University Rotterdam, Rotterdam, The Netherlands

**Keywords:** Monkeypox, Epidemic, Artificial Intelligence, Deep learning, Explainable Artificial Intelligence, LIME, Grad-cam

## Abstract

**Background:**

In May 2022, the World Health Organization (WHO) European Region announced an atypical Monkeypox epidemic in response to reports of numerous cases in some member countries unrelated to those where the illness is endemic. This issue has raised concerns about the widespread nature of this disease around the world. The experience with Coronavirus Disease 2019 (COVID-19) has increased awareness about pandemics among researchers and health authorities.

**Methods:**

Deep Neural Networks (DNNs) have shown promising performance in detecting COVID-19 and predicting its outcomes. As a result, researchers have begun applying similar methods to detect Monkeypox disease. In this study, we utilize a dataset comprising skin images of three diseases: Monkeypox, Chickenpox, Measles, and Normal cases. We develop seven DNN models to identify Monkeypox from these images. Two scenarios of including two classes and four classes are implemented.

**Results:**

The results show that our proposed DenseNet201-based architecture has the best performance, with Accuracy = 97.63%, F1-Score = 90.51%, and Area Under Curve (AUC) = 94.27% in two-class scenario; and Accuracy = 95.18%, F1-Score = 89.61%, AUC = 92.06% for four-class scenario. Comparing our study with previous studies with similar scenarios, shows that our proposed model demonstrates superior performance, particularly in terms of the F1-Score metric. For the sake of transparency and explainability, Local Interpretable Model-Agnostic Explanations (LIME) and Gradient-weighted Class Activation Mapping (Grad-Cam) were developed to interpret the results. These techniques aim to provide insights into the decision-making process, thereby increasing the trust of clinicians.

**Conclusion:**

The DenseNet201 model outperforms the other models in terms of the confusion metrics, regardless of the scenario. One significant accomplishment of this study is the utilization of LIME and Grad-Cam to identify the affected areas and assess their significance in diagnosing diseases based on skin images. By incorporating these techniques, we enhance our understanding of the infected regions and their relevance in distinguishing Monkeypox from other similar diseases. Our proposed model can serve as a valuable auxiliary tool for diagnosing Monkeypox and distinguishing it from other related conditions.

## Background

Monkeypox is a re-emerging zoonotic disease caused by the Monkeypox virus, which belongs to the Poxviridae family, Chordopoxvirinae subfamily, and Orthopoxvirus genus [1]. The virus was first identified in monkeys in 1958 [2] and later found to infect humans in the Democratic Republic of the Congo (DRC) in 1970 [3]. Common initial symptoms of Monkeypox include headache, fever, backache, muscle aches, swollen lymph nodes, and fatigue. Within the first three days of experiencing these symptoms, most infected individuals develop a rash or sores, initially appearing on the face and then rapidly spreading centrifugally to other parts of the body [4].

The spread of the virus had been limited to a few African countries for a considerable period. However, as of the time of writing this article (December 19, 2022), the world was experiencing a global outbreak of the Monkeypox virus. There had been 82,809 confirmed cases reported in 110 countries. In response to this concerning situation, the World Health Organization (WHO) convened an “emergency meeting” on May 20, 2022, to address the escalating cases of the Monkeypox virus. The WHO was deliberating whether to declare the outbreak officially. Furthermore, due to a significant increase in cases, the Centers for Disease Control (CDC) in the United States raised its Monkeypox alert level to “Level 2” on June 6, 2022 [5]. The CDC noted that, as of now, there are no specific treatments available for Monkeypox infection [6]. However, it is worth mentioning that the Food and Drug Administration (FDA) recently approved a Monkeypox vaccine.

As the number of Monkeypox cases continued to rise, countries around the world were implemented various preparations, initiatives, and measures to mitigate the spread of the virus. These efforts included implementing lockdown measures in Belgium, the United States procuring 500,000 doses of the smallpox vaccine, Canada offering vaccination to high-risk groups, French and Danish health authorities advocating for vaccine distribution to adults affected by the virus, Germany recommending vaccinations for high-risk populations, and the United Kingdom advising self-isolation for all individuals infected with the virus [7].

Over the past two decades, significant progress has been made in the development of modern Machine Learning (ML) algorithms, particularly Deep Learning (DL). This progress has been facilitated by the availability of large databases, improved computational power, and increased accessibility to advanced technologies. As a result, Artificial Intelligence (AI) and ML have transitioned from experimental laboratory concepts to practical and applicable technologies in various commercial domains. One sector that has witnessed substantial growth is the application of ML techniques in healthcare. DL has emerged as a prominent player in the field of health informatics, offering distinct advantages in feature extraction and data classification [8].

DL models typically employ a larger number of hidden neurons and layers compared to traditional neural network architectures. This design choice is driven by the availability of vast amounts of raw data during the training phase, enabling the use of more neurons. DL approaches are based on representation learning, which involves constructing nonlinear modules layer by layer to achieve higher levels of representation. Each layer transforms the representation from one form to the next, ultimately resulting in a more abstract representation, thus facilitating the automatic generation of a feature set [9, 10]. In the field of health informatics, the automatic generation of a feature set without human intervention offers significant advantages. Medical image processing is a prominent area where DL has been successfully applied. Among the various DL architectures, Convolutional Neural Networks (CNNs) are commonly used for medical image processing due to their proficiency in computer vision tasks and their ability to leverage Graphics Processing Units (GPUs) [11]. CNNs have found application in various areas such as cancer detection, survival prediction, and the prediction of Coronavirus Disease 2019 (COVID-19) outcomes [12–15]. In recent years, there has been a significant surge in the integration of AI in clinical domains. DL has exhibited remarkable advancements in performance. Various Deep Neural Network (DNN) architectures, such as VGG, ResNet, Inception, AlexNet, GoogLeNet, MobilNet, and ShuffleNet, have been employed for the classification of Monkeypox images [16–18].

Despite their promising performance, a critical concern lies in the limited understanding and interpretability of these models’ decision-making processes. This lack of transparency poses a challenge to gaining trust and acceptance among clinicians. Clinicians often hesitate to fully rely on AI models due to their “black box” nature, where the internal workings and reasoning behind predictions are not readily explainable. To address this issue and establish clarity and certainty, research efforts have focused on developing methods for interpreting and explaining the performance of AI models. These methods aim to unveil the decision-making mechanisms employed by AI models and identify the influential factors that contribute to their predictions [19]. By employing such interpretability techniques, clinicians can gain deeper insights into how AI models arrive at their predictions. This enhanced understanding fosters trust and confidence in the reliability of AI systems, bridging the gap between advanced AI technologies and real-world clinical applications. Some researchers have also emphasized the interpretability and explainability of DL models in the context of Monkeypox. To achieve this, a few studies have employed two approaches, namely Local Interpretable Model-Agnostic Explanations (LIME) and Gradient-weighted Class Activation Mapping (Grad-CAM) [20].

At the time of writing this article, there were only two publicly available datasets that were specifically created for the development of ML and DL models to detect Monkeypox disease [21, 22]. For this study, we utilized the dataset provided by Ahsan et al. [21], which includes skin lesion images of Monkeypox, Chickenpox, and Measles diseases. Considering the available datasets, two scenarios have been explored for Monkeypox detection: multi-class classification, where each class represents a specific skin lesion disease, and two-class classification, such as distinguishing Monkeypox from other diseases.

Despite the existing literature on the detection of Monkeypox disease using DL models, our literature review, along with a recent systematic literature review [20], has identified certain areas that warrant further research:

1. It has been observed that most published studies have focused on developing one of the previously mentioned approaches. Additionally, the two-class approach, specifically distinguishing Monkeypox from other diseases, has emerged as more prevalent in the literature [20].
2. Most studies have primarily reported accuracy as the main metric for evaluating the performance of their models. This trend is also evident in [20], where accuracy is the sole metric utilized across all included studies. However, it has been well-established that accuracy alone may not provide a comprehensive evaluation of model performance. In light of this, the Receiver Operating Characteristic (ROC) curve, which offers a measurement based on the surface, has been proposed as an alternative evaluation metric [23].
3. The importance of model interpretability has been recognized, but only a limited number of studies have addressed this aspect [16, 24]. Understanding how models arrive at their predictions is crucial for ensuring trust, transparency, and effective decision-making in clinical settings.

Therefore, further research is needed to explore alternative approaches, consider diverse evaluation metrics, and delve into the interpretability of DL models in the detection of Monkeypox disease. By addressing these gaps, we can enhance the effectiveness and reliability of these models for real-world applications.

### Objectives and contributions

In this study, we utilized the previously mentioned publicly available dataset [21] and performed preprocessing techniques to prepare the data for analysis. Subsequently, we implemented seven DL models that leverage pre-trained capabilities to diagnose Monkeypox disease based on skin lesion images from patients. To enhance the performance of these models, we conducted experiments and introduced modifications to the standard architecture, incorporating five dense layers. Additionally, we explored two scenarios: the two-class scenario and the four-class scenario. In the two-class scenario, images were categorized into Monkeypox and non-Monkeypox classes, while the four-class scenario involved Monkeypox, Chickenpox, Measles, and Normal classes. These scenarios allowed us to develop robust models and facilitate a comprehensive analysis of the data.

To ensure the optimal performance of the models, we conducted rigorous hyperparameter optimization and evaluated their performance using eight different evaluation metrics. Additionally, to enhance interpretability and provide explanations for the models’ results, we employed LIME and Grad-Cam techniques [19]. The contributions of this paper can be summarized as follows:

1. Development of seven modified DNNs, specifically designed for the detection of Monkeypox, considering both two-class and four-class scenarios.
2. Evaluation of model performance and generalization capabilities using eight different performance metrics, providing a comprehensive understanding of the effectiveness of the models.
3. Utilization of LIME and Grad-Cam techniques to enhance the interpretability of the models, allowing for a better understanding of the factors influencing the models’ decisions.
4. Comparative analysis with previous studies that employed similar scenarios, demonstrates the superior performance of our proposed model in terms of the F1-Score metric for both scenarios. Additionally, we reported ROC curves and Area Under the Curve (AUC) values for all models, further validating their performance.

## Materials and methods

### Dataset and preprocessing

The increasing global incidence of Monkeypox infection has captured the attention of researchers, leading to efforts aimed at exploring early detection methods for this contagious disease. A crucial aspect of these endeavors involves leveraging the potential of ML techniques to accurately identify and distinguish Monkeypox from other similar diseases. To initiate this task, researchers have commenced data collection and the construction of datasets as an initial step.

The dataset of this study consists of 43 Monkeypox, 47 Chickenpox, 27 Measles, and 54 normal images. In order to standardize the images, we resized them to 128 × 128 pixels. To enhance the dataset for more robust training, we employed augmentation techniques to increase the number of samples. The augmentation process involved the following parameters: rotation range = 45, rescale = 1/255, zoom range = 0.15, height shift range = 0.25, width shift range = 0.25, shear range = 0.25, channel shift range = 25, vertical flip = True, and horizontal flip = True. Table 1 provides a brief overview of the dataset.

**Table 1:** Dataset description

Disease	Dataset original samples	Dataset samples after augmentation
Monkeypox	43	430
Chickenpox	47	470
Measles	27	270
Normal	54	540
Total	171	1710

### Deep models

For the classification task, seven CNN architectures were implemented, namely InceptionResNetV2, InceptionV3, ResNet152V2, VGG16, VGG19, Xception, and DenseNet201 [25, 26].

InceptionResNetV2 is a CNN architecture that was released in 2015. It combines two networks: the inception architecture and residual connections. This network comprises 164 layers and has approximately 56 million parameters [25].

InceptionV3, released in 2016, is an optimized version of InceptionV1. It consists of 42 layers and has fewer than 25 million parameters [26].

ResNet152V2 is a residual neural network and the second version of ResNet152. It was developed in 2016 and includes 152 layers with approximately 1.7 million parameters [26].

VGG16 and VGG19 are CNNs introduced in 2015, both consisting of 138 million parameters. These networks differ in the number of weight layers, with VGG16 having 16 weight layers and VGG19 having 19 weight layers [26].

The Xception architecture, released in 2017, incorporates residual connections and a subset of convolution layers. It is 71 layers deep and comprises approximately 20 million parameters [25].

DenseNet201, proposed in 2017, is a CNN that directly connects all layers. It consists of 201 layers and approximately 20 million parameters [26].

**Fig. 1:**
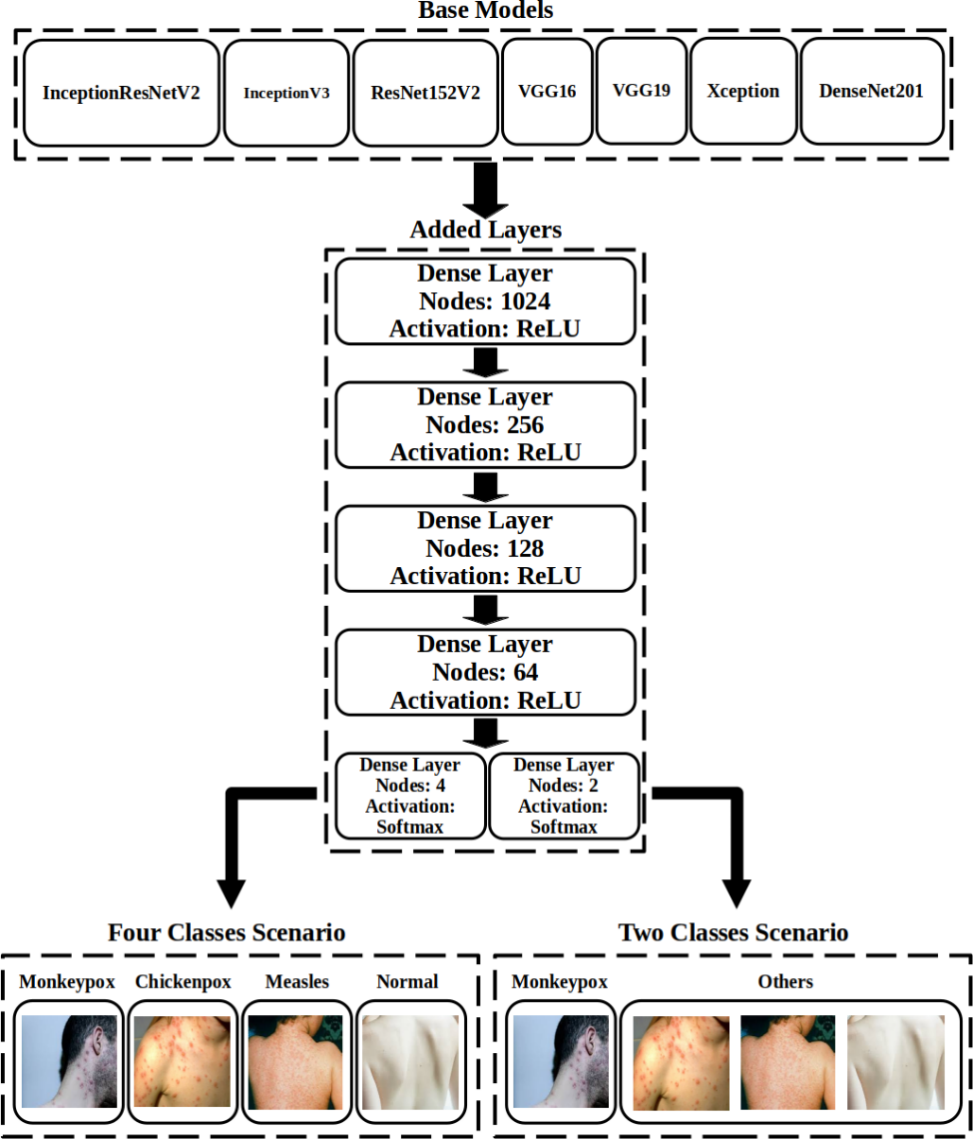
Method diagram

These DNN models have often been trained on large, publicly available datasets such as ImageNet, enabling them to recognize various image properties. Leveraging pre-trained networks offers advantages in terms of training speed and accuracy when applied to new tasks, such as image classification. This is because pre-trained models can transfer significant image features that have already been learned to the new task, eliminating the need to learn them again from scratch. To further enhance the results, we incorporated five Dense layers into all DNN models. The methodology diagram is depicted in Fig. 1.

### Models tuning

The grid search technique was employed to identify the optimal values for hyperparameters. This method involves systematically searching through a predefined set of hyperparameter values to determine the best combination for a specific model. Additionally, 5-fold cross-validation was implemented to ensure the models’ generalizability and obtain more reliable performance estimates. In each iteration of cross-validation, 80% of the data was used for training, while the remaining 20% was reserved for testing. It is important to note that a small portion of the training set was also utilized for hyperparameter tuning during each iteration to find the best hyperparameter values.

Based on our experimental findings, we incorporated four dense, fully connected layers following the pre-trained models to diagnose the disease in this study. The number of units for these four layers was determined through a grid search. Additionally, at the end of the proposed models’ architecture, another dense fully connected layer with four units and a softmax activation function was added to determine the class label in the four-class scenario. Similarly, this layer had two units to determine the class in the two-class scenario. This process is shown in Fig. 1 and the optimal values for hyperparameters are presented in Table 2.

**Table 2:** Best values for the models’ hyperparameters

Model	Learning Rate	Batch Size	Number of epochs	Loss Function	Activation Function
InceptionResNetV2	0.00001	10	10	SGD	Tanh
InceptionV3	0.001	12	20	Adam	ReLU
ResNet152V2	0.001	10	15	SGD	ReLU
VGG16	0.00001	10	15	SGD	ReLU
VGG19	0.0001	10	15	SGD	ReLU
Xception	0.0001	12	15	Adam	Tanh
DenseNet201	0.0001	8	10	Adam	ReLU

### Evaluation metrics

After applying cross-validation and identifying the optimal hyperparameters, the performance of all models was assessed using ROC and AUC. In addition to ROC and AUC, six other metrics were employed to evaluate the models’ performance: Accuracy, F1-Score, Precision or Positive Predictive Value (PPV), Negative Predictive Value (NPV), Specificity, and Sensitivity (Recall) [14, 27].

### Model explainability

LIME is a commonly used technique for interpreting and explaining AI models. It offers a transparent approach to comprehensively assess the performance of AI black box models. LIME allows for the local interpretation and analysis of individual examples, enabling a better understanding of the model’s decision-making process. This method is applicable to various types of data, including images, text, and tabular data, making it a versatile tool for model interpretation [19].

Grad-CAM was also employed in this study for result interpretation. Grad-CAM utilizes the gradients of a target concept that flow into the final convolutional layer of a model. It generates a coarse localization map that highlights the significant regions in an image, indicating the areas that are important for predicting the concept of interest. By visualizing these important regions, Grad-CAM provides insights into which parts of the image contribute most to the model’s decision-making process [28].

## Results

As mentioned earlier, this study implemented seven DNNs and evaluated their performance in two scenarios. After applying hyperparameter optimization and 5-fold cross-validation, the performance of all models was assessed using the introduced metrics. The results for both four-class and two-class approaches are presented below.

### Four-class scenario

**Fig. 2:**
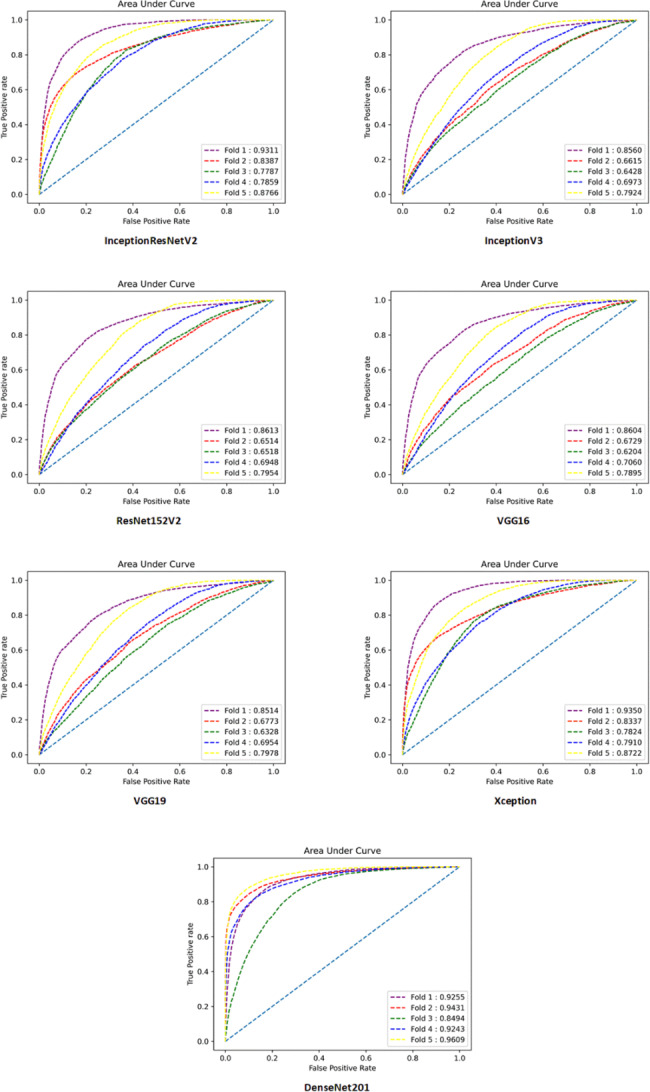
AUC diagram of all folds for developed models in a four-class scenario

**Fig. 3:**
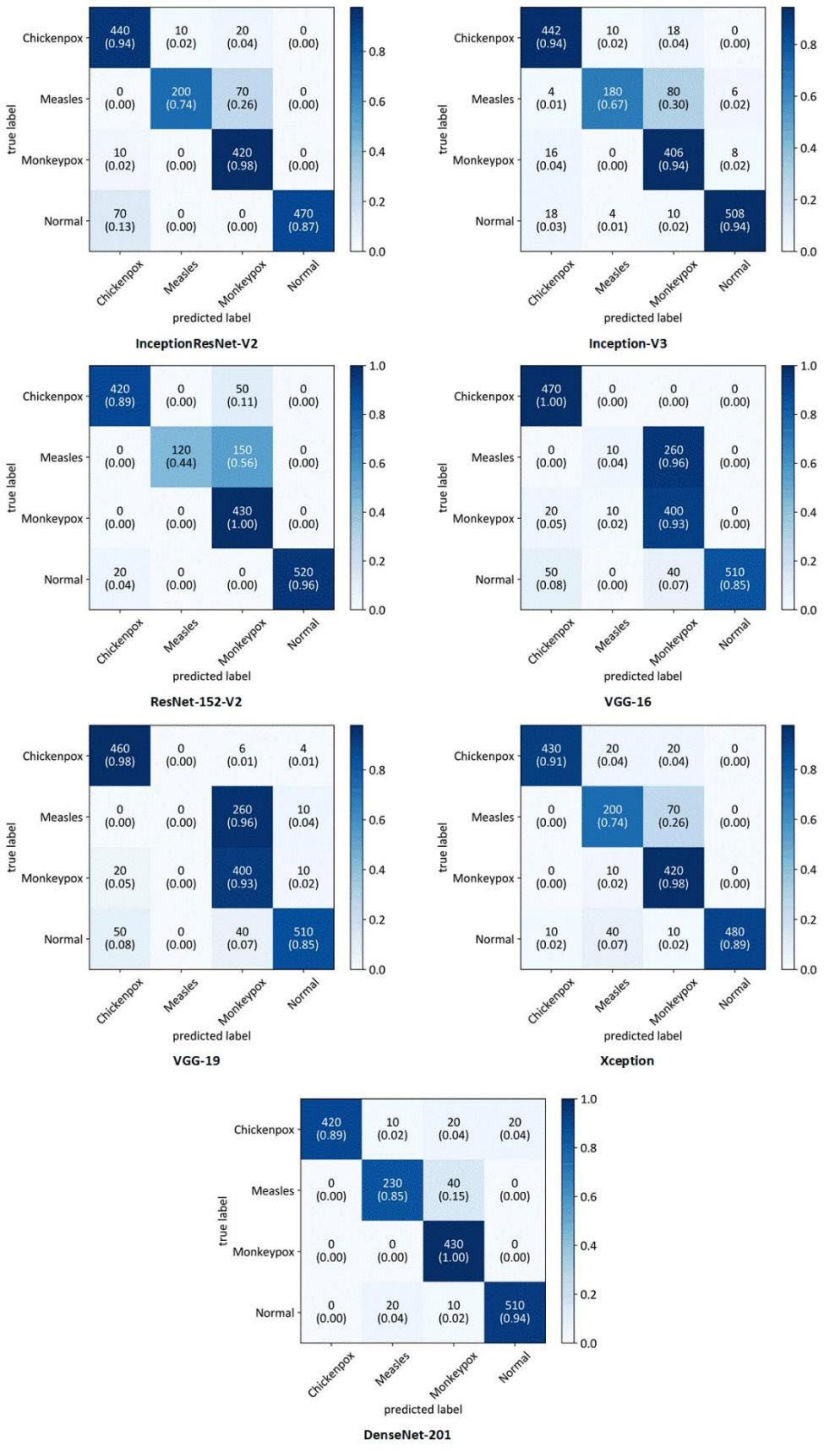
Average confusion matrices of all folds for developed models in a four-class scenario

Table 3; Fig. 2 present a comprehensive overview of the performance of all the developed DL models, considering various performance metrics. It is evident that DenseNet201 exhibited the highest performance across all metrics. There are some advantages for DenseNet architecture making this DNN a robust model that can outperform other architectures. DenseNet architecture addresses a critical issue in high-level neural networks where information tends to dissipate before reaching its final destination due to the significant distance between input and output layers. DenseNet was specifically designed to overcome this challenge, resulting in improved accuracy and performance. The unique connectivity patterns in DenseNet facilitate the flow of information throughout the network, enabling enhanced information propagation and feature extraction [29]. Additionally, Table 4 provides a detailed breakdown of the performance of DenseNet201 for each fold. This allows for a more comprehensive understanding of how effectively the model assigned the correct class to each sample. Furthermore, to gain insights into the overall classification performance of all developed models, Fig. 3 showcases the confusion matrices for each model. These matrices provide a visual representation of the model’s ability to accurately classify samples into their respective classes.

**Fig. 4:**
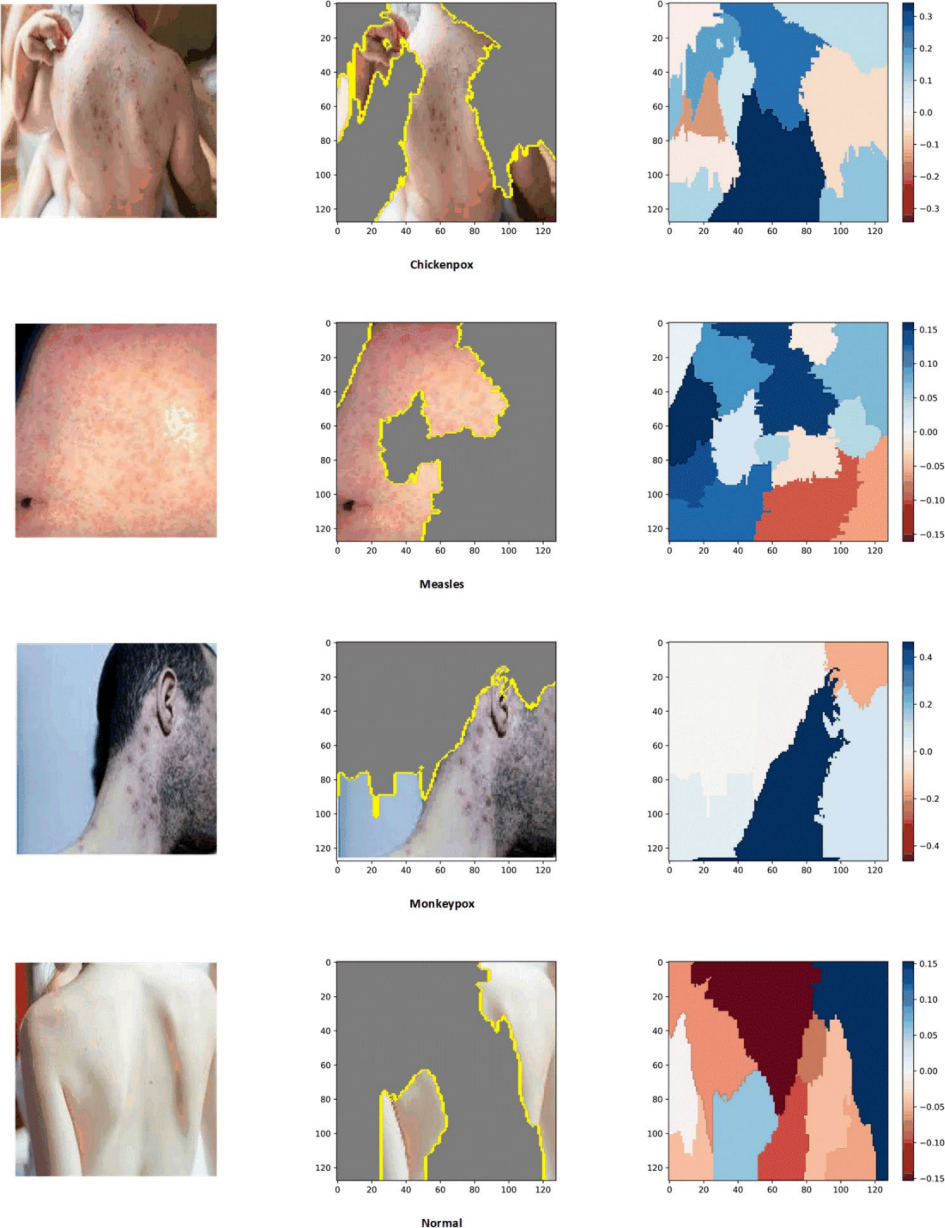
Explainability and interpretability of the best model (DenseNet201) with LIME in a four-class scenario

**Fig. 5:**
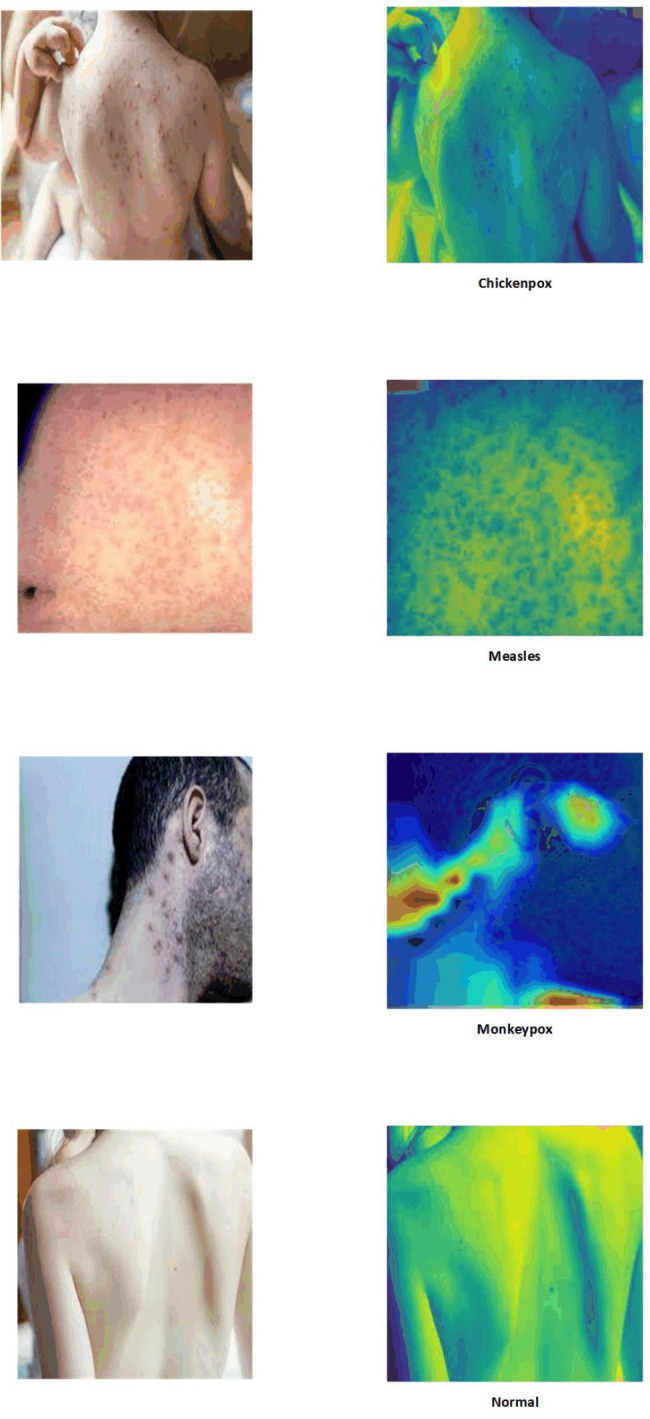
Explainability and interpretability of the best model (DenseNet201) with Grad-Cam in a four-class scenario

Lastly, for the purpose of interpretability and identifying image regions that are closely associated with the four conditions, LIME and Grad-Cam techniques were employed. These techniques offer valuable insights by highlighting the specific regions within the images that contribute significantly to the classification of each condition. This aids in understanding the decision-making process of the models and provides valuable visual explanations for their predictions. The results of this experiment are depicted in Figs. 4 and 5, respectively. As seen both techniques could identify those regions which are more associated with poxes and diseases. It is important to note that the blue color signifies a more severe condition, while the red color indicates less affected areas.

Furthermore, the ability to identify sensitive areas in the decision-making process provides a valuable tool for clinicians, enabling them to gain a better and clearer understanding of the AI black box model.

**Table 3:** Performance of DNN models for four-class (Monkeypox, Chickenpox, Measles, Normal) approach

Model	Accuracy (%)	F1-Score (%)	NPV (%)	PPV (%)	Specificity (%)	Sensitivity (%)	AUC (%)
InceptionResNetV2	94.48	88.85	96.47	89.95	96.27	88.95	84.22
InceptionV3	94.74	87.93	96.64	89.98	96.09	88.90	73.00
ResNet152V2	94.17	88.39	96.89	90.72	95.62	87.13	73.09
VGG16	88.78	71.10	93.66	77.09	91.79	75.56	72.98
VGG19	87.20	67.21	90.86	74.19	90.40	62.10	73.09
Xception	95.02	88.41	96.78	88.94	95.83	88.61	84.29
DenseNet201	**95.18**	**89.61**	**97.10**	**90.73**	**96.50**	**89.82**	**92.06**

**Table 4:** Fold-wise performance of the best model (DenseNet201) in a four-class approach

Fold	Accuracy (%)	F1-Score (%)	NPV (%)	PPV (%)	Specificity (%)	Sensitivity (%)	AUC (%)
Fold 1	96.59	92.95	97.82	93.00	97.59	92.98	92.55
Fold 2	93.48	86.24	96.10	88.87	95.21	86.55	94.31
Fold 3	95.92	91.10	97.50	91.64	97.08	91.23	84.94
Fold 4	94.45	88.15	96.56	89.36	96.07	88.30	92.43
Fold 5	95.50	89.65	97.52	90.82	96.57	90.06	96.09
*Average*	**95.18**	**89.61**	**97.10**	**90.73**	**96.50**	**89.82**	**92.06**

### Two-class scenario

**Fig. 6:**
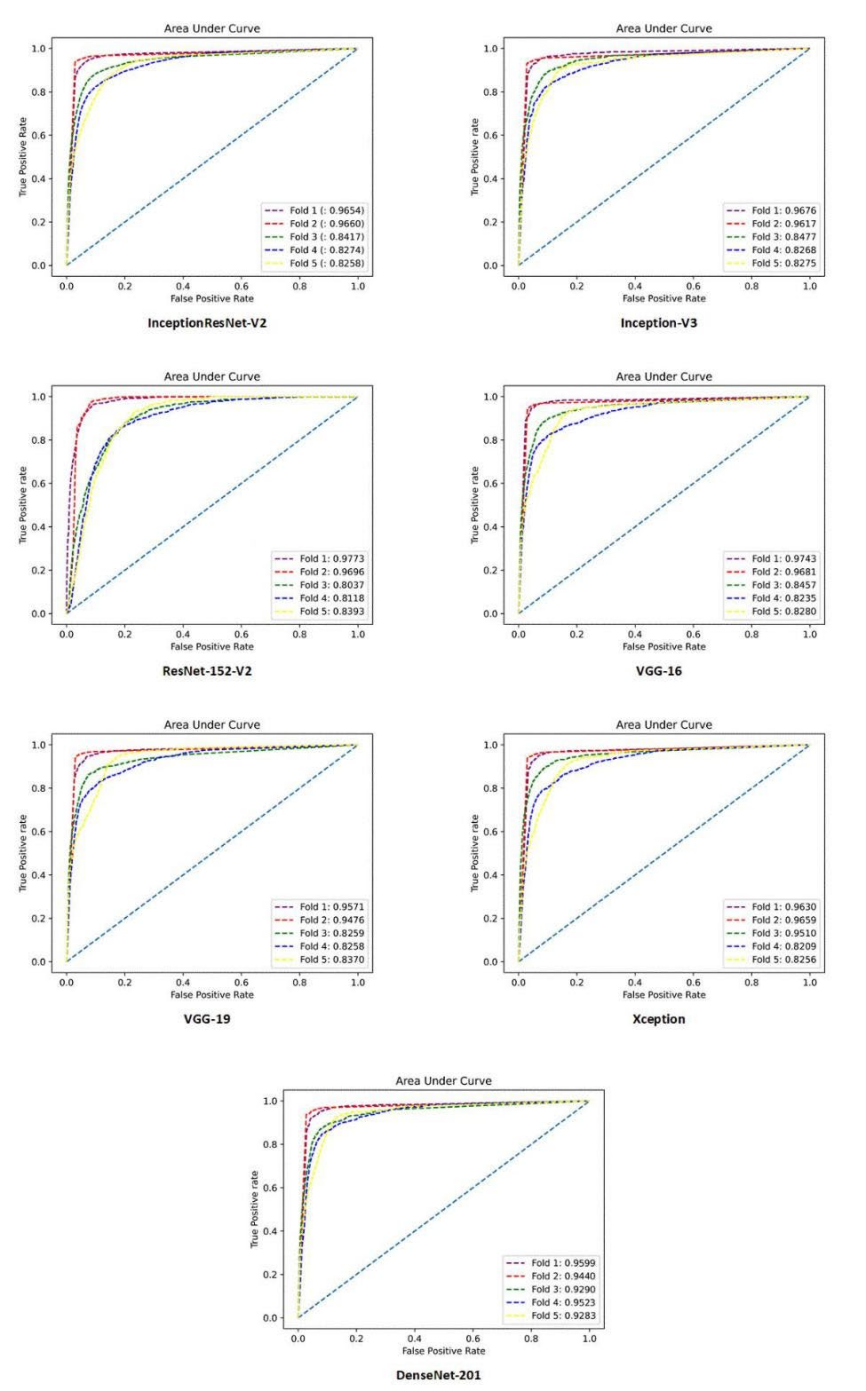
AUC diagram of all folds for developed models in a two-class scenario

**Fig. 7:**
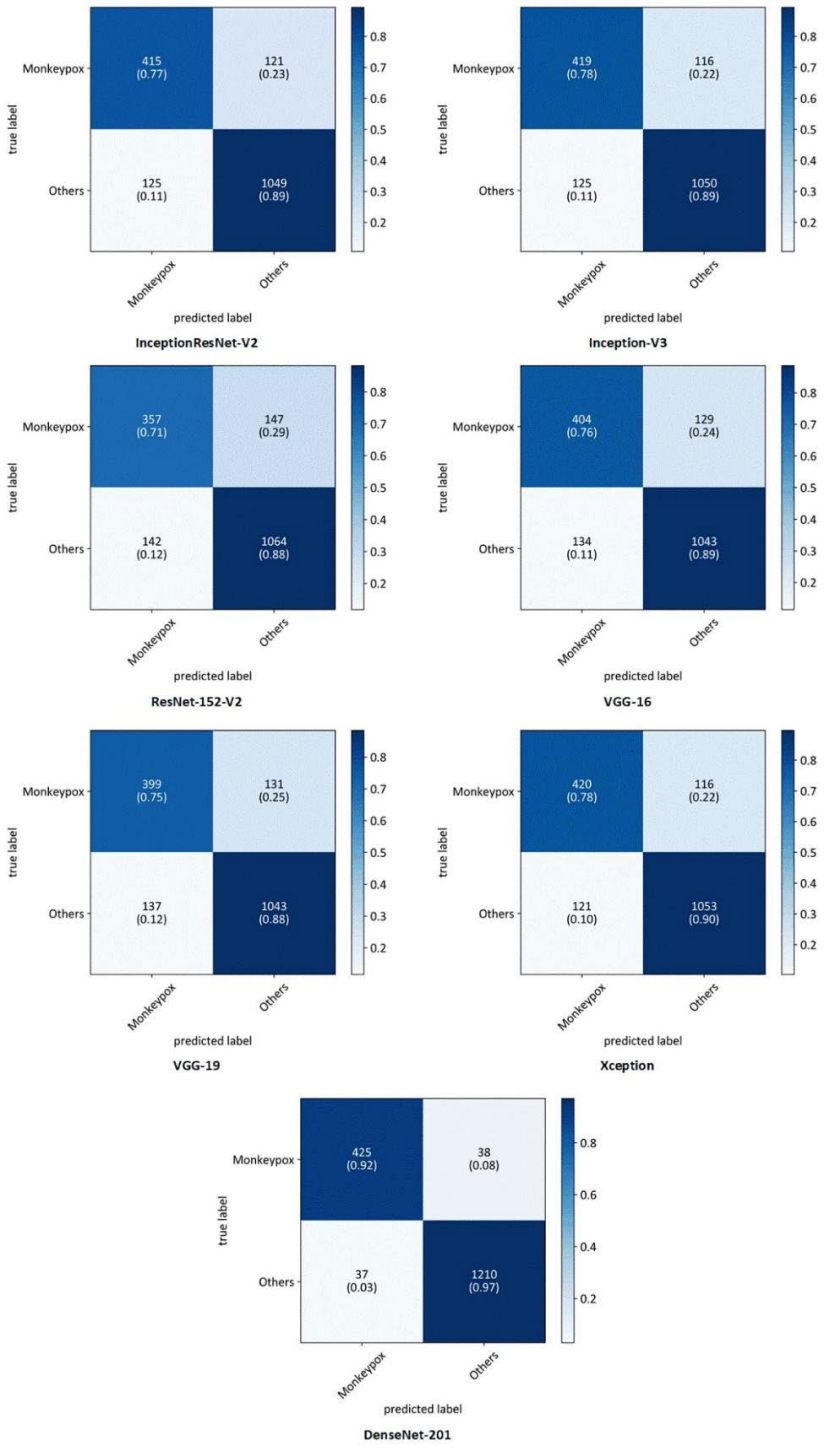
Average confusion matrices of all folds for developed models in a two-class scenario

In the second scenario, our main objective was to accurately detect Monkeypox disease. For this purpose, we employed the “one versus all” classification approach. Monkeypox images were classified as the first class, while Chickenpox, Measles, and Normal images were grouped as the second class. The performance of all models was evaluated using various metrics, as presented comprehensively in Table 5; Fig. 6. Additionally, we calculated and visualized the confusion matrices for all models in Fig. 7. Once again, DenseNet201 consistently demonstrated the best performance across all metrics. To provide a deeper understanding of its performance, we present the results of each fold for DenseNet201 in Table 6.

It is worth noting that the average performance of DenseNet201 in this scenario surpassed that of the previous scenario. This suggests that Monkeypox images possess unique characteristics and attributes that enable the DNN to identify them more effectively in the “one versus all” classification approach. This observation highlights the discriminative nature of the features associated with Monkeypox, allowing the model to differentiate them from other conditions with greater accuracy.

**Fig. 8:**
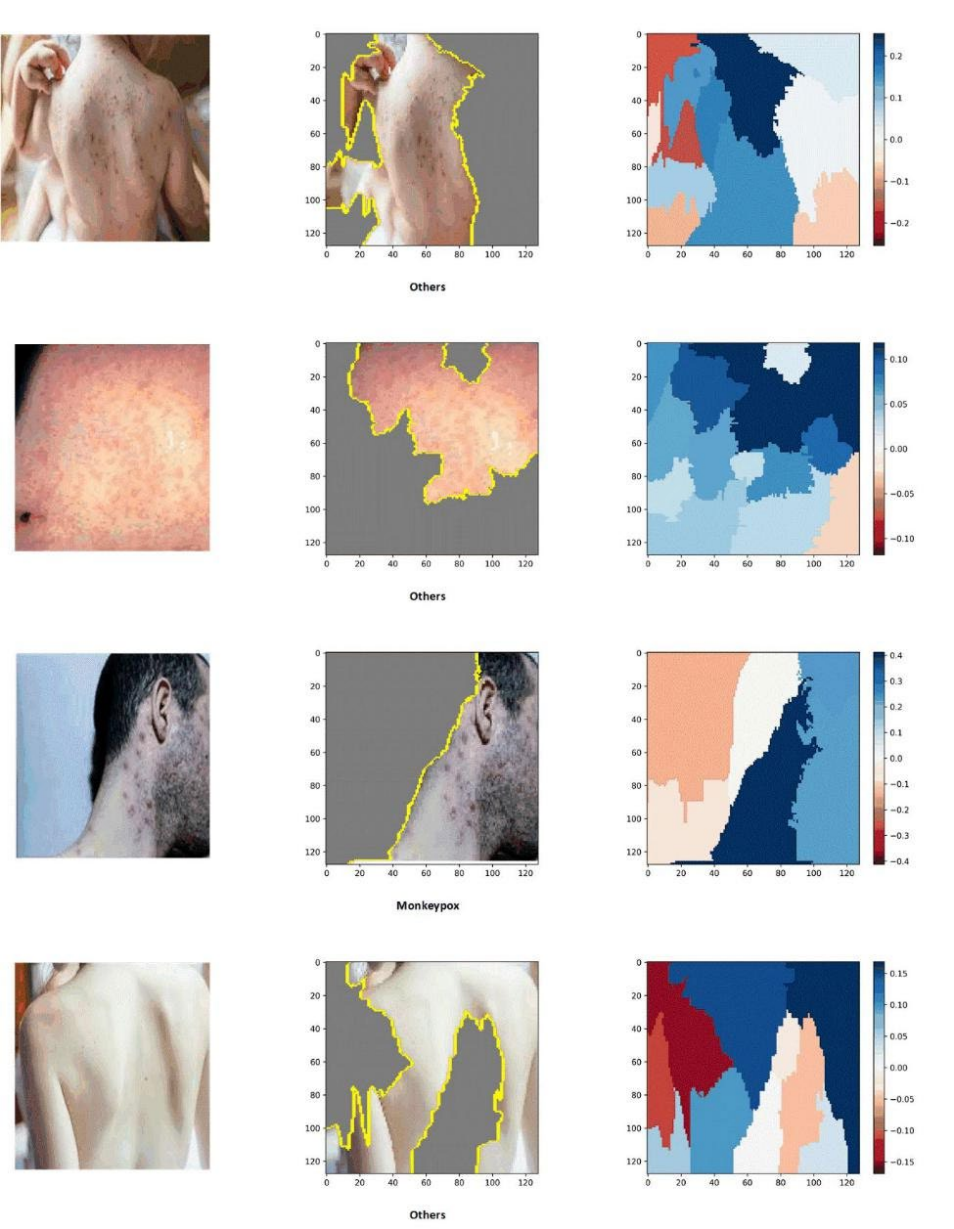
Explainability and interpretability of the best model (DenseNet201) with LIME in a two-class scenario

**Fig. 9:**
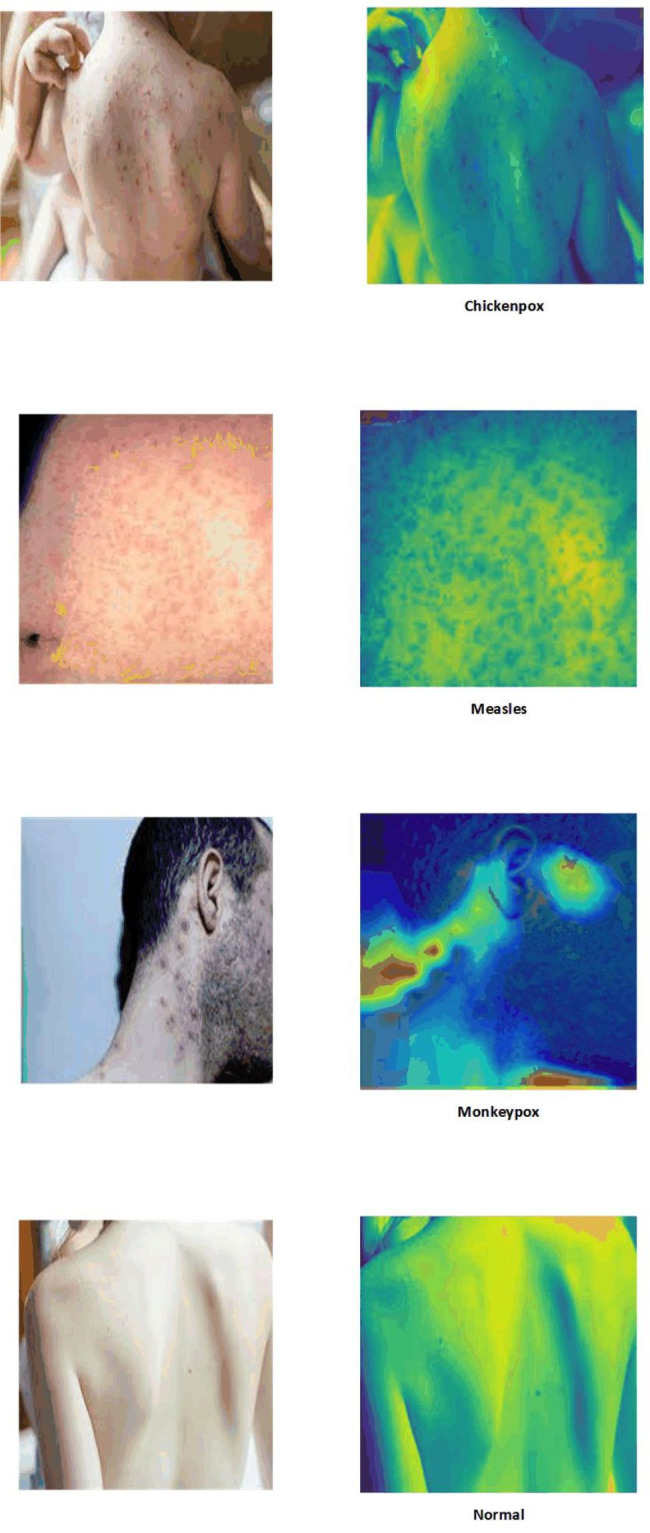
Explainability and interpretability of the best model (DenseNet201) with Grad-Cam in a two-class scenario

To enhance the interpretability of the models’ outputs, we applied LIME and Grad-Cam techniques, and the results are illustrated in Figs. 8 and 9. Both LIME and Grad-Cam techniques demonstrate their proficiency in identifying the crucial areas within the images that play a significant role in the classification of poxes and disorders.

**Table 5:** Performance of DNN models for two-class (Monkeypox versus others)

Model	Accuracy (%)	F1-Score (%)	NPV (%)	PPV (%)	Specificity (%)	Sensitivity (%)	AUC (%)
InceptionResNetV2	85.64	78.06	87.59	78.40	87.57	78.02	86.52
InceptionV3	85.90	79.34	87.73	79.69	87.72	79.21	88.62
ResNet152V2	84.26	70.69	86.84	71.63	86.81	70.30	88.03
VGG16	85.11	75.10	87.30	75.14	87.28	75.06	88.79
VGG19	84.85	73.32	87.18	75.19	87.13	73.25	87.66
Xception	85.99	79.70	87.79	80.07	87.77	79.69	90.52
DenseNet201	**97.63**	**90.51**	**98.89**	**89.96**	**98.47**	**91.08**	**94.27**

**Table 6:** Fold-wise performance of the best model (DenseNet201) in a two-class approach

Fold	Accuracy (%)	F1-Score (%)	NPV (%)	PPV (%)	Specificity (%)	Sensitivity (%)	AUC (%)
Fold 1	98.31	91.68	99.23	90.30	98.89	93.10	95.99
Fold 2	96.81	85.14	99.03	79.69	97.41	91.40	94.40
Fold 3	97.38	86.95	98.59	86.61	98.50	87.30	92.90
Fold 4	97.82	89.41	99.11	86.96	98.47	92.00	95.23
Fold 5	97.83	88.87	98.52	91.25	99.08	86.60	92.83
*Average*	**97.63**	**90.51**	**98.89**	**89.96**	**98.47**	**91.08**	**94.27**

**Table 7:** Comparison of our study with previous studies in a four-class (Monkeypox, Chickenpox, Measles, and Normal) scenario

Study	Model	Accuracy (%)	Precision (%)	Recall (%)	F1-Score (%)	AUC (%)
Abdelhamid et al. [24]	AlexNet, VGG19, GoogLeNet, ResNet50	**98.8**	-	-	**-**	**-**
Situala et al. [16]	13 DNNs	87.13	85.44	85.47	85.4	-
Our study	Modified 7 DNNs	95.18	**90.73**	**89.82**	**89.61**	**92.06**

**Table 8:** Comparison of our study with previous studies in a two-class (Monkeypox versus others) scenario

Study	Model	Accuracy (%)	Precision (%)	Recall (%)	F1-Score (%)	AUC (%)
Ahsan et al. [17]	VGG16	78	75	75	75	**-**
Ali et al. [22]	VGG16, ResNet50, InceptionV3, Ensemble	82.96	87	83	84	**-**
Sahin et al. [18]	EfficientNetb0, MobilNetV2	91.11	86.36	**95**	90.48	-
Haque et al. [36]	5 DNNs	83.89	90.70	89.10	90.11	-
Saleh et al. [37]	ML and DNN models	**98.48**	**91.11**	89	-	-
Our study	Modified 7 DNNs	97.63	89.96	89.96	**91.08**	**94.27**

## Discussion

A recent systematic literature review revealed a more than 10-fold increase in Monkeypox cases over the past five decades [30]. This highlights the growing significance of Monkeypox as a public health concern. The COVID-19 pandemic has further emphasized the need for preparedness before such crises occur. Ongoing debates suggest that COVID-19 might not be the last pandemic in our lifetime, underscoring the importance of proactive measures and readiness for future health emergencies. These arguments stress the importance of proactive preparation for similar situations. It is crucial for researchers across various disciplines, such as medicine, public health, and data science, to collaborate and develop robust infrastructures before a disease escalates into a global crisis, akin to a pandemic. At the time of writing this article, Monkeypox had garnered significant attention from health authorities worldwide, raising concerns about its potential to become the next pandemic if timely and appropriate actions are not taken [31]. Furthermore, it is worth mentioning that WHO believes that Monkeypox has not gone away and there is much possibility it comes back within the Europe region during this spring and summer [32].

ML and DL play an undeniable role in disease detection, prediction, and assisting clinicians in decision-making. AI-driven clinical solutions, especially in radiological and histopathological image analysis, have achieved notable success with advancements in computer vision. These solutions provide analytical support and aid in the diagnosis, empowering physicians without replacing them [33].

In this study, we developed seven modified DL models specifically designed for diagnosing Monkeypox diseases. The dataset included samples from diseases that exhibit skin lesions similar to Monkeypox, such as Chickenpox and Measles. Accurately distinguishing Monkeypox from other diseases that present with similar skin poxes is crucial. Misdiagnosis can occur as multiple diseases can manifest similar skin lesions. For instance, Monkeypox and Smallpox, both associated with Orthopoxviruses, produce similar skin lesions. However, during the prevalence of Smallpox, there were no documented instances of Monkeypox. This could be due to the focus primarily being on Smallpox, leading to a similarity in appearances or an assumption that Smallpox was the cause in the absence of laboratory evidence of the etiologic agent [34].

Including samples from various diseases that cause poxes allowed us to develop more accurate models for diagnosing and distinguishing between these similar conditions. By expanding the dataset to encompass different diseases with similar symptoms, our models can provide improved accuracy in identifying and differentiating specific conditions.

In our study, we examined two scenarios for diagnosing Monkeypox: two classes and multi classes. The results revealed that DenseNet201 exhibited the highest performance across all eight performance metrics for both scenarios. In addition, our study demonstrated that DenseNet201 performed better in the two classes approach compared to the multi classes approach. These results reinforce the superiority of DenseNet201 as an outstanding performer in accurately detecting Monkeypox disease. The higher average performance in a two-class scenario validates the model’s capability to leverage unique characteristics specific to Monkeypox images, contributing to enhanced diagnostic accuracy and highlighting the importance of considering distinct features of different diseases in classification tasks.

The lack of interpretability in DNN poses legal, ethical, and trust challenges, as they are often perceived as “black-box” or difficult to understand. This lack of transparency can lead to issues and raise doubts among clinicians and patients regarding the reliability of DNN models. To address this challenge, techniques like LIME and Grad-Cam have been introduced and have shown promising results, validated by clinicians, in interpreting DNN outputs [35]. Therefore, in this study, we applied these techniques to perform image segmentation and highlight the regions that are most relevant to the infections, aiming to improve the interpretability of our models.

Furthermore, we conducted a comparative analysis of our best-performing model with previous studies that employed similar settings [16–18, 20, 22, 24, 36, 37]. These studies were categorized into two groups: four-class and two-class scenarios. The results of these comparisons are presented in Tables 7 and 8, respectively. Upon examining Table 7, it is evident that our modified DenseNet201 model outperformed the other studies in terms of precision, recall, F1-Score, and AUC (except for accuracy). Notably, our study is the only one that reported the AUC metric. The inclusion of the AUC metric further enhances the comprehensive evaluation of our model’s performance and based on Mandrekar et al.‘s study [38], the performance of our proposed model can be described as “outstanding”.

Table 8 shows the comparison of our study with similar studies using a two-class approach. As shown, different studies had the best performance for different performance metrics. Among them, our proposed DNN outperformed other studies for F1-Score and AUC metrics. Similar to the previous scenario, DenseNet201 has greater value than 90 for AUC which is considered as “outstanding” performance [38].

In conclusion, our best proposed model exhibited superior performance compared to previous studies in both scenarios, specifically in terms of the F1-Score and AUC metrics. The performance of our model warrants its classification as “outstanding” [38] in both scenarios, indicating its potential applicability for the detection of Monkeypox using skin images. Furthermore, our experiments demonstrated that the trained model can successfully detect a Monkeypox image in just 65 seconds. This finding suggests the feasibility of using our system as a real-time application, providing timely and efficient detection of Monkeypox cases.

Overall, the performance and real-time capabilities of our proposed system hold promising implications for the field of Monkeypox detection and contribute to the advancement of AI-based diagnostic tools in healthcare.

Despite the promising results, our study has some limitations. The main limitation of this study is related to the dataset. The current dataset is not clinically approved and only contains skin images. However, collecting other features like laboratory tests seems essential to have robust models that can work in real practice.

## Conclusion

This study aimed to explore the potential of DL models in detecting Monkeypox using skin images. The dataset used in this study consisted of 1710 samples from four classes: Monkeypox, Chickenpox, Measles, and Normal cases. Seven DL models, including InceptionResNetV2, InceptionV3, ResNet152V2, VGG16, VGG19, Xception, and DenseNet201, were developed for both multi-class and binary-class approaches. Cross-validation and hyperparameters optimization techniques were employed to ensure the generalizability of the models.

The performance of the developed models was evaluated using eight metrics. Our results consistently showed that DenseNet201 outperformed the other models in terms of all metrics in both scenarios. While different studies may have outperformed others in specific evaluation metrics, our modified DenseNet201 exhibited superior performance in terms of F1-Score for both scenarios. It is worth noting that the use of the AUC metric, which is considered a benchmark in the healthcare domain, was not reported in previous studies. However, our best model achieved an AUC greater than 90, indicating an “outstanding” performance. This underscores the potential of our proposed model as an auxiliary tool for clinicians in the quick and early diagnosis of Monkeypox disease.

To enhance trust and transparency, we incorporated LIME and Grad-Cam techniques, which provide interpretability and explainability of the decision-making process in AI models. These techniques allowed us to identify the infected regions and their significance in the diagnosis of various diseases using skin images.

In our future work, we plan to validate the performance of our proposed model using different datasets of clinical images to demonstrate the generalizability of our approach. Additionally, we aim to explore the feasibility of developing decision support software that can be integrated into clinical practice for the timely detection of Monkeypox.

## Data Availability

The dataset used in this study can be downloaded from the website at https://github.com/ASorayaie/MPD-DNN.

## Abbreviations

WHO: World Health Organization
DRC: Democratic Republic of the Congo
CDC: Centers for Disease Control
FDA: Food and Drug Administration
ML: Machine Learning
DL: Deep Learning
AI: Artificial Intelligence
CNN: Convolutional Neural Network
COVID-19: Coronavirus Disease 2019
DNN: Deep Neural Network
LIME: Local Interpretable Model-Agnostic Explanations
Grad-Cam: Gradient-weighted Class Activation Mapping
PPV: Positive Predictive Value
NPV: Negative Predictive Value
ROC: Receiver operating characteristic
AUC: Area Under the Curve

## References

[1] McCollum AM, Damon IK (2014). Human monkeypox. Clin Infect Dis.

[2] von Magnus P, Andersen EK, Petersen KB, Birch-Andersen A (1959). A pox‐like disease in cynomolgus monkeys. Acta Pathologica Microbiologica Scandinavica.

[3] Breman JG, Steniowski MV, Zanotto E, Gromyko AI, Arita I (1980). Human monkeypox, 1970-79. Bull World Health Organ.

[4] Vaughan A et al. “Human-to-human transmission of monkeypox virus, United Kingdom, October 2018,” *Emerging infectious diseases*, vol. 26, no. 4, p. 782, 2020. 10.3201/eid2604.191164 PMC7101111 32023204

[5] SR K. “CDC raises Monkeypox travel alert to level 2.” [Online]. Available: https://www.forbes.com/sites/suzannerowankelleher/2022/06/07/cdc-raises-Monkeypox-travel-alert-to-level-2/?sh=67c264f83f93.

[6] “CDC. Treatment information for healthcare professionals. Centers for Disease Control and Prevention.” [Online]. Available: https://www.cdc.gov/poxvirus/Monkeypox/clinicians/treatment.html.

[7] Sukhdeo S, Mishra S, Walmsley S (2022). Human monkeypox: a comparison of the characteristics of the new epidemic to the endemic disease. BMC Infect Dis.

[8] Naemi A, Schmidt T, Mansourvar M, Naghavi-Behzad M, Ebrahimi A, Wiil UK. Machine learning techniques for mortality prediction in emergency departments: a systematic review. BMJ Open. Nov. 2021;11(11):e052663. 10.1136/bmjopen-2021-052663. 10.1136/bmjopen-2021-052663 PMC8565537 34728454

[9] Ravì D (2016). Deep learning for health informatics. IEEE J biomedical health Inf.

[10] Miotto R, Wang F, Wang S, Jiang X, Dudley JT (2018). Deep learning for healthcare: review, opportunities and challenges. Brief Bioinform.

[11] Navamani TM. “Efficient deep learning approaches for health informatics,” in Deep learning and parallel computing environment for bioengineering systems, Elsevier, 2019, 123–37.

[12] Azar AS et al. “Lightweight Method for the Rapid Diagnosis of Coronavirus Disease 2019 from Chest X-ray Images using Deep Learning Technique,” in *2021 IEEE Nuclear Science Symposium and Medical Imaging Conference (NSS/MIC)*, 2021, pp. 1–5. 10.1109/NSS/MIC44867.2021.9875630.

[13] Moncada-Torres A, van Maaren MC, Hendriks MP, Siesling S, Geleijnse G (2021). Explainable machine learning can outperform Cox regression predictions and provide insights in breast cancer survival. Sci Rep.

[14] Rikan SB, Azar AS, Ghafari A, Mohasefi JB, Pirnejad H (2022). COVID-19 diagnosis from routine blood tests using artificial intelligence techniques. Biomed Signal Process Control.

[15] Chen HJ, Mao L, Chen Y, Yuan L, Wang F, Li X, Cai Q, Qiu J, Chen F. Machine learning-based CT radiomics model distinguishes COVID-19 from non-COVID-19 pneumonia. BMC Infect Dis. 2021 Dec;21(1):1–3. 10.1186/s12879-021-06614-6. 10.1186/s12879-021-06614-6 PMC8424152 34496794

[16] Sitaula C, Shahi TB (2022). Monkeypox virus detection using pre-trained deep learning-based approaches. J Med Syst.

[17] Ahsan MM, Uddin MR, Farjana M, Sakib AN, Al Momin K, Luna SA. “Image Data collection and implementation of deep learning-based model in detecting Monkeypox disease using modified VGG16,” *arXiv preprint arXiv:2206.01862*, 2022.

[18] Sahin VH, Oztel I, Yolcu Oztel G (2022). Human monkeypox classification from skin lesion images with Deep Pre-trained Network using Mobile Application. J Med Syst.

[19] Palatnik de Sousa I, M. Maria Bernardes Rebuzzi Vellasco, and, Costa da Silva E. “Local interpretable model-agnostic explanations for classification of lymph node metastases,” *Sensors*, vol. 19, no. 13, p. 2969, 2019. 10.3390/s19132969 PMC6651753 31284419

[20] Chadaga K (2023). Application of Artificial Intelligence Techniques for Monkeypox: a systematic review. ” Diagnostics.

[21] Ahsan MM, Uddin MR, Luna SA. “Monkeypox Image Data collection,” *arXiv preprint arXiv:2206.01774*, 2022.

[22] Ali SN, Ahmed M, Paul J, Jahan T, Sani SM, Noor N, Hasan T. Monkeypox skin lesion detection using deep learning models: a feasibility study. arXiv preprint arXiv:2207.03342. 2022 Jul 6.

[23] Novaković J, Dj (2017). Evaluation of classification models in machine learning. Theory and Applications of Mathematics & Computer Science.

[24] Abdelhamid AA et al. “Classification of monkeypox images based on transfer learning and the Al-Biruni Earth Radius Optimization algorithm.“ Mathematics 10.19 (2022): 3614.

[25] Phankokkruad M. “COVID-19 pneumonia detection in chest X-ray images using transfer learning of convolutional neural networks,” in *Proceedings of the 3rd international conference on data science and information technology*, 2020, pp. 147–152.

[26] Chai J, Zeng H, Li A, Ngai EWT (2021). Deep learning in computer vision: a critical review of emerging techniques and application scenarios. Mach Learn Appl.

[27] Safari S, Baratloo A, Elfil M, Negida AS (2015). Part 2: positive and negative predictive values of diagnostic tests. Archives of Academic Emergency Medicine.

[28] Selvaraju RR et al. “Grad-CAM: Why did you say that?.“ arXiv preprint arXiv:1611.07450 (2016).

[29] Huang G et al. “Densely connected convolutional networks.“ Proceedings of the IEEE conference on computer vision and pattern recognition. 2017.

[30] Bunge EM (2022). The changing epidemiology of human monkeypox—A potential threat? A systematic review. PLoS Negl Trop Dis.

[31] Kumar N, Acharya A, Gendelman HE, Byrareddy SN. “The 2022 outbreak and the pathobiology of the monkeypox virus,” J Autoimmun, p. 102855, 2022. 10.1016/j.jaut.2022.102855 PMC9534147 35760647

[32] An mpox resurgence in the European Region this spring. and summer? To prevent that, key measures must continue [Internet]. Who.int. [cited 2023 May 28]. Available from: https://www.who.int/europe/news/item/17-05-2023-an-mpox-resurgence-in-the-european-region-this-spring-and-summer--to-prevent-that--key-measures-must-continue.

[33] “Broadband Commission. Reimagining Global Health through Artificial Intelligence. The Roadmap to AI Maturity.”; 2020.

[34] Reynolds MG, Carroll DS, Karem KL (2012). Factors affecting the likelihood of monkeypox’s emergence and spread in the post-smallpox era. Curr Opin Virol.

[35] Kumarakulasinghe NB, Blomberg T, Liu J, Leao AS, Papapetrou P. “Evaluating local interpretable model-agnostic explanations on clinical machine learning classification models,” in *2020 IEEE 33rd International Symposium on Computer-Based Medical Systems (CBMS)*, 2020, pp. 7–12.

[36] Haque Md et al. “Classification of human monkeypox disease using deep learning models and attention mechanisms.“ arXiv preprint arXiv:221115459 (2022).

[37] Saleh AI, Asmaa H (2023). Rabie. “Human monkeypox diagnose (HMD) strategy based on data mining and artificial intelligence techniques. Comput Biol Med.

[38] Mandrekar JN (2010). Receiver operating characteristic curve in diagnostic test assessment. J Thorac Oncol.

